# Endoscopic submucosal dissection followed by chemoradiotherapy for superficial esophageal cancer: choice of new approach

**DOI:** 10.1186/s13014-018-1195-7

**Published:** 2018-12-14

**Authors:** Gen Suzuki, Hideya Yamazaki, Norihiro Aibe, Koji Masui, Naomi Sasaki, Daisuke Shimizu, Takuya Kimoto, Atsushi Shiozaki, Osamu Dohi, Hitoshi Fujiwara, Takeshi Ishikawa, Hirotaka Konishi, Yuji Naito, Eigo Otsuji, Kei Yamada

**Affiliations:** 1 Department of Radiology, Kyoto Prefectural University Graduate School of Medical Science, 465 Kajiicho Kawaramachi Hirokoji, Kamigyo-ku, Kyoto, 602-8566 Japan; 2 Department of Digestive Surgery, Kyoto prefectural university Graduate School of Medical Science, 465 Kajiicho Kawaramachi Hirokoji, Kamigyo-ku, Kyoto, 602-8566 Japan; 3 Department of Gastroenterology and Hepatology, Kyoto prefectural university Graduate School of Medical Science, 465 Kajiicho Kawaramachi Hirokoji, Kamigyo-ku, Kyoto, 602-8566 Japan

**Keywords:** Superficial esophageal cancer, Endoscopic submucosal dissection, Chemoradiotherapy, Esophagectomy, T1b

## Abstract

**Background:**

The standard treatment for superficial esophageal cancer (SEC) involving muscularis mucosal (T1a-MM) or submucosal (T1b) invasion has been the surgical resection of the esophagus. However, esophagectomy with extended lymph node dissection is highly invasive. Recent reports have shown that endoscopic submucosal dissection (ESD) followed by chemoradiotherapy (CRT) has promising results and might become a new therapeutic approach. This retrospective study aimed to elucidate the efficacy and safety of this new treatment.

**Methods:**

Patients with clinical stage T1b tumor without apparent metastasis treated with ESD followed by CRT from 2014 to 2017 (the CRT group) were included. The outcomes on disease-free survival (DFS) of this group were compared with those of consecutive patients in a historical control group who underwent ESD followed by esophagectomy (the esophagectomy group) between 2008 and 2015.

**Results:**

Of 32 patients analyzed, 16 were in the CRT group and 16 with similar stage cancer were in the esophagectomy group. Radiotherapy was completed in all patients, and the incidence of grade ≥ 3 nonhematologic adverse events was 6%. The 2-year overall survival rates were 100%, and locoregional control was achieved in all patients in the CRT group, and the 2-year DFS rates were 88 and 100% for the CRT and esophagectomy groups, respectively, without significant differences.

**Conclusions:**

Our data confirmed our new approach as being safe and effective for locoregional control and may provide a nonsurgical treatment option for patients with clinical stage T1b tumors.

## Background

The incidence of superficial esophageal cancer (SEC) is increasing, particularly in Asian countries, including Japan where the screening for upper digestive tract cancers is common [1, 2]. For several years, the standard treatment for SEC with submucosal invasion has been esophagectomy with extended lymph node dissection. Although the 3-year survival rate of patients with submucosal tumors surgically treated is > 80%, disadvantages include the substantial risk of major surgical complications, small-but-real risk of perioperative death, a recovery period of several months, and the potential for long-term swallowing problems [3, 4].

Endoscopic submucosal dissection (ESD) is a minimally-invasive procedure with high curability for patients with SEC without metastasis (T1N0M0) and has been recently established as a standard treatment for SEC following encouraging early reports from Japan [5]. Despite the excellent local tumor control after ESD, the presence of submucosal (T1b) or muscularis mucosal (T1a-MM) invasion with lymphovascular invasion increases the risk for lymph node metastases; therefore, these patients cannot be treated with ESD alone [6–8]. Thus, additional curative treatments after ESD are indispensable.

The efficacy of concurrent chemoradiotherapy (CRT), which is much less invasive than esophagectomy, has recently been demonstrated [9–11]. However, the main limitations of definitive CRT are local failures. ESD is conducted to completely remove or reduce the size of superficial tumors, which reduces the risk of local failure by approximately 20% [9]. Although standard additional treatments after ESD have not yet been established, CRT would be a new alternative therapeutic approach to esophagectomy in patients with T1a-MM and T1b tumors [9, 12].

We evaluated the combined treatment of ESD followed by CRT (the CRT group) in patients with clinical stage T1b tumors, which may have a substantial risk of recurrence. Clinical outcomes of this group were then compared with those of patients in a historical control group who previously underwent esophagectomy after ESD (the esophagectomy group) at our hospital.

## Methods

### Patients

The study was approved by the institutional review board of the Kyoto Prefectural University of Medicine (Permission code: ERB-C-1104). Between January 2014 and April 2017, consecutive patients who refused conventional esophagectomy but underwent ESD followed by CRT were included. All patients had clinical stage T1b tumors. For the evaluation of clinical T1b tumor, magnifying endoscopy was performed in all patients in addition to standard endoscopy, chromoendoscopy with Lugol’s iodine solution, and CT.

Patients were excluded if there was any evidence of metastasis based on imaging studies using contrast-enhanced computed tomography (CT) obtained from neck to abdomen and positron emission tomography/CT (PET/CT) of the entire body.

The indication for additional treatments after ESD was decided as per the modified Japanese guidelines for esophageal cancer [13]. Briefly, the criteria for additional treatments were decided on the basis of the following findings in the pathological specimens: muscularis mucosae (T1a-MM) invasion; positive lymphovascular invasion, including lymphatic or vascular invasion; infiltration pattern C (INF C), indicating cancer nests exhibiting infiltrative growth; and an unclear and droplet infiltration pattern (DI) [14, 15]. Invasion to the submucosa (T1b) and/or a positive resection margin also were regarded as indications for additional treatment. Written informed consent for ESD followed by CRT was obtained from all patients.

### Pathological evaluation and classification

Resected specimens were microscopically examined by at least two experienced pathologists and evaluated according to the Japanese Classification of Esophageal Cancer, 11th edition [16]. Briefly, tumors with invasion to the mucosal, submucosal, and muscularis propria were defined as T1a, T1b, and T2, respectively. T1a and T1b tumors were further divided into three subtypes according to the extent of invasion as T1a-EP, mucosal epithelium; T1a-LPM, lamina propria mucosae; and T1a-MM, muscularis mucosa and T1b-SM1, upper-third stratum of the submucosal layer; T1b-SM2, middle-third stratum of the submucosal layer; and T1b-SM3, lower-third stratum of the submucosal layer.

### Chemotherapy

A follow-up endoscopy was performed 1–2 months after ESD. CRT was initiated after confirming the scarring of ESD-induced ulcers. The chemotherapy regimen included continuous 5-fluorouracil (FU, 1000 mg/m^2^/d on days 1–4 and 29–32) and cisplatin (CDDP, 75 mg/m^2^/d on days 1 and 29).

### Radiotherapy

**Fig. 1:**
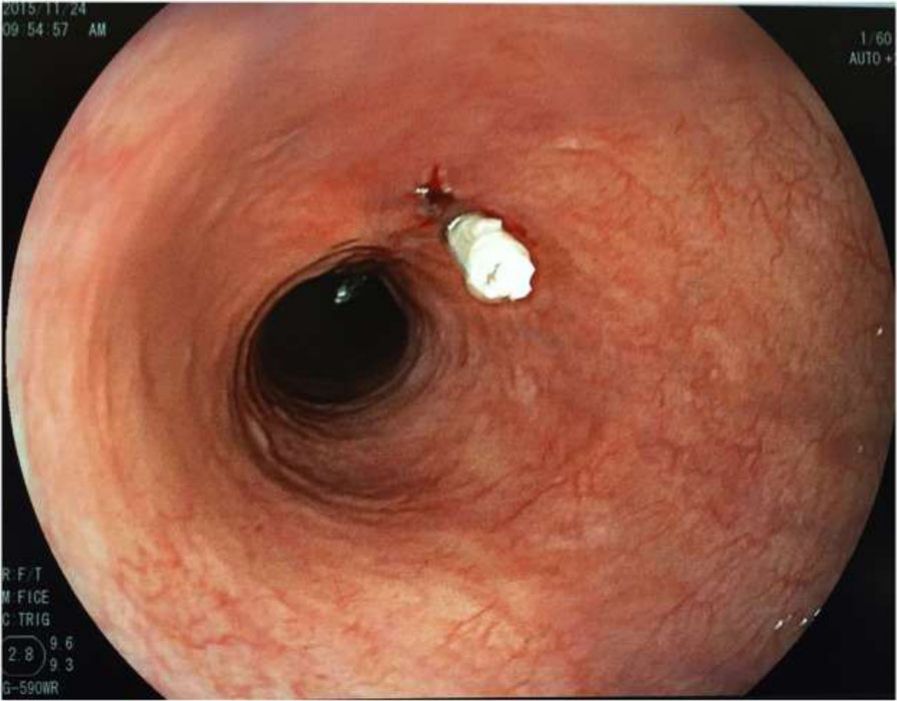
Endoscopy shows metal clip marked at the distal and proximal ends of the tumor bed

Megavoltage photon beam radiotherapy was concurrently initiated with systemic chemotherapy. All patients underwent CT simulations before treatment. The tumor bed was marked with a clip before obtaining a planning CT scan (Fig. 1). The location of the tumor bed was defined on the basis of the scarring tissue created by ESD.

**Fig. 2:**
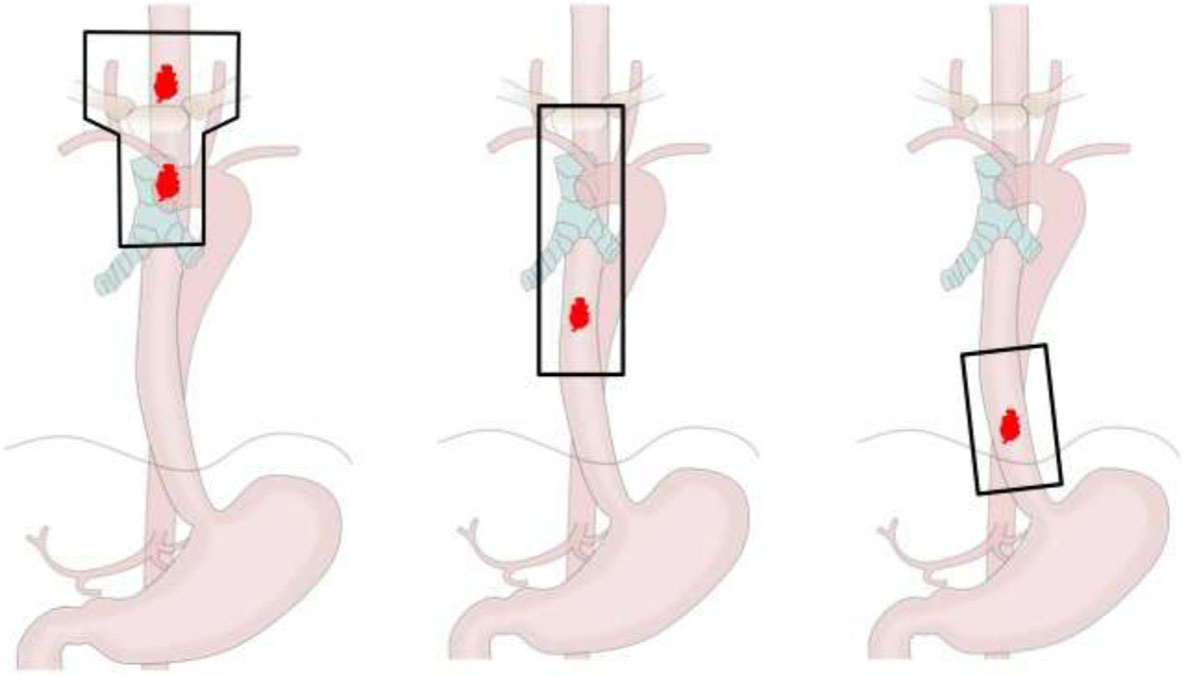
Irradiation fields for esophageal carcinoma according to the location of the tumor

Three-dimensional conformal radiotherapy (2 Gy per day for 5 d per week) with a linear accelerator (6 or 10 MV) was applied to the treatment. A total dose of 40 Gy was administered to the initial clinical target volume (CTV1) in patients with negative resection margins to prevent lymph node recurrence (Fig. 2). CTV1s included bilateral supraclavicular and mediastinal lymph node regions to the bifurcation of the trachea for upper esophageal cancers; superior mediastinum and 2 cm below the distal end of the tumor bed marked with a clip oriented along the esophagus for middle thoracic tumors; and a tumor bed with 2-cm craniocaudal margins oriented along the esophagus for lower thoracic tumors. Large elective nodal areas were not included.

In patients with positive resection margins, the tumor bed with 2-cm craniocaudal margins as CTV2 was irradiated with a total dose of 50 Gy in the boost plans (2 Gy per day for 5 d per week). The planning target volume (PTV) was defined as the CTV plus 1-cm margins in all directions in the initial and boost plans. The dose was prescribed to the isocenter in the middle of the planned target volume. Treatment fields were adjusted using a multileaf collimator to reduce the maximal dose to the spinal cord to < 40 Gy. Anteroposterior and oblique four-port field method was routinely used to reduce the cardiac radiation damage in middle and lower thoracic tumors.

### Follow-up and evaluation

All patients were followed-up to detect local recurrence or distant metastasis every 3–4 months during the first 2 years and every 6 months thereafter, with blood tests, upper gastrointestinal endoscopy with iodine staining, and CT of the neck/chest/abdomen. Follow-up data were obtained from electronic medical records. Locoregional recurrence was defined as the recurrence of the primary tumor or metastases to the regional lymph node observed on endoscopy or CT.

### Historical control

We compared the treatment results of the CRT group with those of a previously reported historical control group of 16 patients who underwent esophagectomy after ESD (the esophagectomy group) from 2008 to 2015 at our hospital [17].

### Statistical analysis

Baseline characteristics of treatment groups were compared using the Mann–Whitney *U* test for continuous variables and the χ^2^ test or Fisher’s exact test for categorical variables. The overall (OS) and disease-free (DFS) survival and locoregional control were calculated using to the Kaplan–Meier method, starting from the day when the initial treatment began. Differences between the groups were estimated using the log-rank test. All statistical analyses were performed using EZR (Saitama Medical Center, Jichi Medical University, Saitama, Japan), which is a graphical user interface for R (The R Foundation for Statistical Computing, Vienna, Austria) and a modified version of the R commander designed to add statistical functions frequently used in biostatistics [18]. In all analyses, *P* < 0.05 was considered significant.

## Results

### Patient characteristics

Sixteen patients (13 males, 81%) were treated with ESD followed by CRT during the study period. The rate of complete follow-up was 100%, median observation period was 24 (range, 12–51) months, and median age was 69 (range, 50–80) years. Twelve (75%) patients had clinical stage T1b tumor. Lymphatic and vascular invasions were observed in 11 (69%) and three (19%) patients, respectively. Five (31%) patients had a positive resection margin (vertical in two and parallel in three). Patients’ characteristics are summarized in Table 1.

**Table 1:** Patient characteristics

Characteristic	Chemoradiotherapy (*n* = 16)	Esophagectomy (*n* = 16)	*P*-value
Median age (range), years	67 (46–87)	64 (51–77)	0.07
Sex, *n*			
Male	13	16	0.23
Female	3	0	
Histological type, *n*			
Squamous cell carcinoma	16	12	0.1
Adenocarcinoma	0	4	
Main tumor location, *n*			
Upper thorax	3	2	0.14
Middle thorax	8	8	
Lower thorax	5	2	
Abdominal	0	4	
ESD-T Stage, *n*			
T1a^a^	4	9	0.15
T1b	12	7	
ESD-ly, *n*			
Positive	11	10	> 0.99
Negative	5	6	
ESD-v, *n*			
Positive	3	4	> 0.99
Negative	13	12	
ESD-INF, *n*			
C	1	2	> 0.99
DI	11	5	0.08
ESD-HM, *n*			
Positive	3	1	0.6
Negative	13	15	
ESD-VM, *n*			
Positive	2	2	> 0.99
Negative	14	14	
Total radiation dose			
40 Gy	12	–	
50 Gy	4	–	

*ESD*, endoscopic submucosal dissection; *ly*, lymphatic invasion; *v*, vascular invasion; *INF*, infiltration; *DI,* droplet infiltration; *HM*, horizontal margin; *VM*, vertical margin

^a^patoholgical stage

### Treatment outcomes

**Fig. 3:**
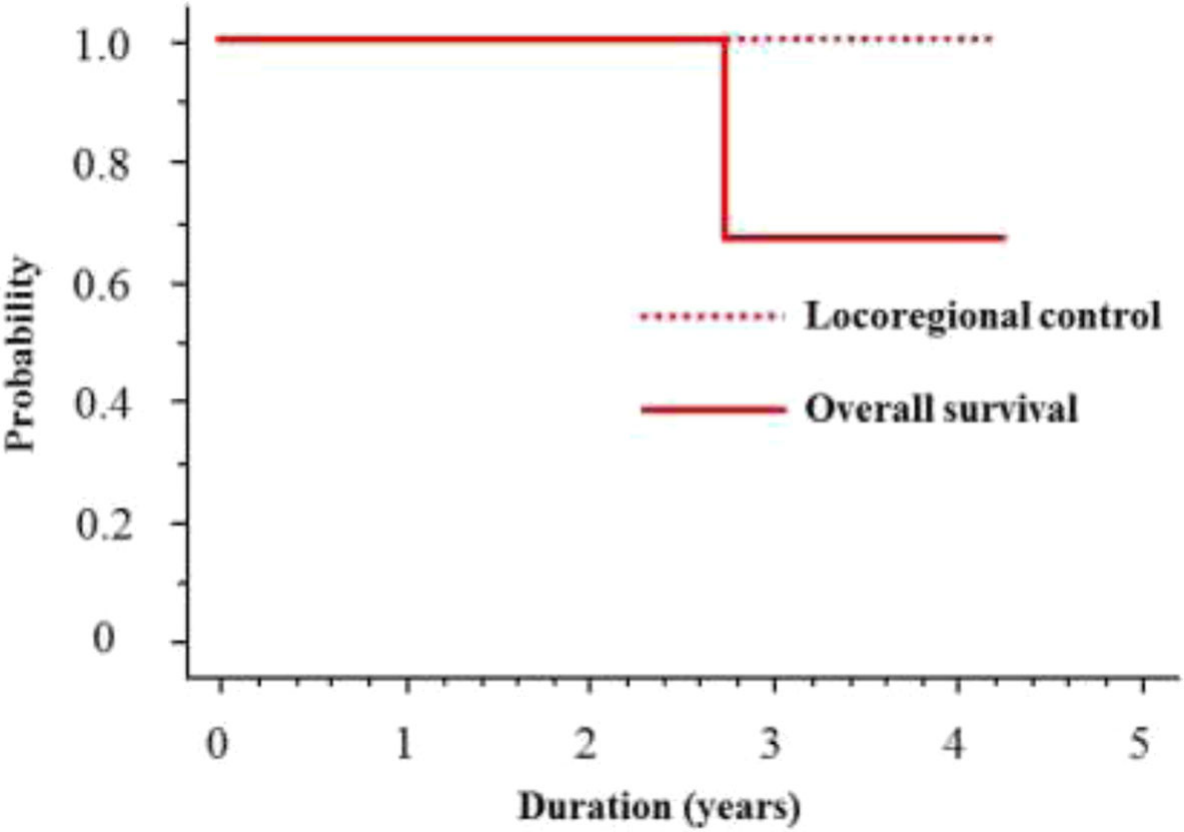
Overall survival (OS) and locoregional control curves for patients treated with ESD + CRT. ESD + CRT: endoscopic submucosal dissection + chemoradiotherapy. The 2-year OS rate was 100%, and locoregional control was achieved in all patients

Radiotherapy was completed in all patients. Four patients refused the second chemotherapy cycle. One patient died of esophageal cancer with distant lymph node recurrence. No other patient died of other causes throughout the study period. The 2-year OS rate was 100%, and locoregional control was achieved in all patients (Fig. 3). During the follow-up, metachronous esophageal lesions occurred in two patients, one in and one out of the irradiation field, which were successfully treated with ESD.

**Fig. 4:**
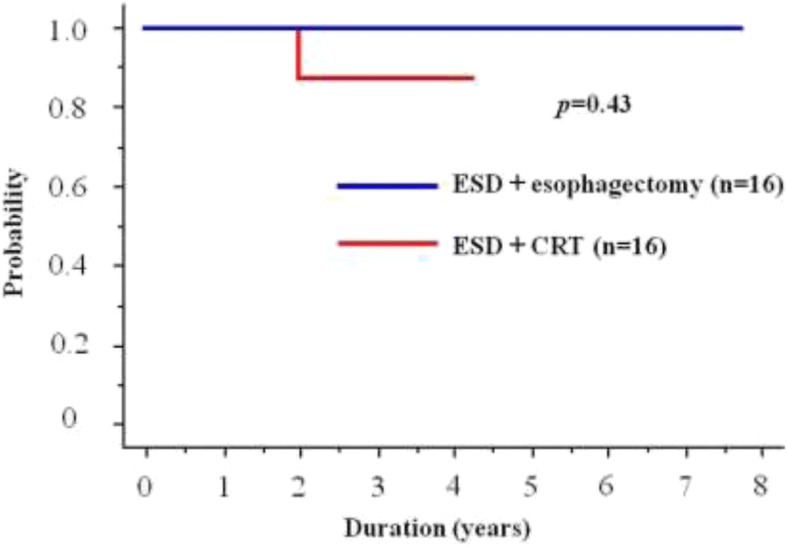
Comparison of disease-free survival (DFS) curves between ESD + CRT and ESD + esophagectomy groups. ESD + CRT: endoscopic submucosal dissection + chemoradiotherapy: ESD + esophagectomy: endoscopic submucosal dissection + esophagectomy. The 2-year DFS rates of the CRT group (88%) was not significantly different (*P* = 0.43) from that of the esophagectomy group (100.0%)

A patient with an SM3 tumor developed distant lymph node recurrence (paraaortic lymphadenopathy). No hematological distant metastasis was observed. The 2-year DFS was 88% (Fig. 4). Table 2 summarizes the pathological examination of ESD specimens and clinical outcomes for patients who underwent additional CRT.

**Table 2:** Pathological findings of patients with SEC who underwent ESD followed by CRT

Patient	Age	Sex	T Stage	ly	v	INF C	DI	HM	VM	Months to disease recurrence (site)
1	69	Male	T1b-SM2	(+)	(−)	(−)	(−)	(−)	(−)	(−)
2	78	Male	T1b-SM2	(−)	(−)	(−)	(−)	(−)	(+)	(−)
3	60	Male	T1b-SM3	(+)	(−)	(−)	(+)	(+)	(−)	24 (paraaortic lymph node)
4	80	Male	T1b-SM2	(−)	(−)	(−)	(−)	(−)	(−)	(−)
5	55	Male	T1a-MM	(+)	(−)	(−)	(+)	(−)	(−)	(−)
6	73	Male	T1b-SM1	(−)	(+)	(+)	(+)	(−)	(−)	(−)
7	65	Male	T1b-SM2	(+)	(−)	(−)	(+)	(−)	(−)	(−)
8	74	Female	T1a-MM	(+)	(+)	(−)	(+)	(−)	(−)	(−)
9	68	Male	T1b-SM1	(+)	(−)	(−)	(+)	(−)	(−)	(−)
10	68	Female	T1b-SM2	(+)	(−)	(−)	(+)	(−)	(−)	(−)
11	71	Male	T1a-MM	(+)	(−)	(−)	(+)	(−)	(−)	(−)
12	80	Male	T1b-SM1	(+)	(−)	(−)	(+)	(+)	(−)	(−)
13	50	Male	T1b-SM2	(−)	(−)	(−)	(−)	(−)	(−)	(−)
14	76	Female	T1b-SM2	(+)	(+)	(−)	(−)	(−)	(+)	(−)
15	68	Male	T1b-SM1	(−)	(−)	(−)	(+)	(+)	(−)	(−)
16	60	Male	T1a-MM	(+)	(−)	(−)	(+)	(−)	(−)	(−)

*SEC*, superficial esophageal cancer; *ESD*, endoscopic submucosal dissection; *CRT,* chemoradiotherapy; *T1a-MM*, tumor invading muscularis mucosae; *T1b-SM1*, tumor invading the upper third of the submucosa; *T1b-SM2*, tumor invading the middle third of the submucosa; *ly*, lymphatic invasion; *v*, vascular invasion; *INF*, infiltration; *DI,* droplet infiltration; *HM*, horizontal margin; *VM*, vertical margin

### Toxicities

Toxicities were scored according to the National Cancer Institute Common Terminology Criteria for Adverse Events (NCI-CTCAE) version 4.0. Grade ≥ 3 adverse events occurred in five (31%) patients, including grade 3 leukopenia in four (25%) and grade 3 esophagitis in one (6%). No patients experienced treatment interruption over 1 week due to acute toxicities. No patients experienced grade ≥ 4 toxicity, radiation pneumonitis, or pericardial effusion. Grade 2 esophageal strictures were observed in two patients during follow-up.

### Additional chemoradiotherapy versus additional esophagectomy (historical control)

The characteristics of both groups are summarized in Table 1. No significant differences were observed in terms of sex, pathological invasion depth, lymphovascular invasion, or the type of infiltration. The 2-year OS rate was 100% for both groups, and the 2-year DFS rates were 88 and 100% for the CRT and esophagectomy groups, respectively (not statistically significant, *P* = 0.43; Fig. 4).

## Discussion

Definitive CRT has recently become a less invasive treatment option for SEC compared with esophagectomy [19]. Although OS after definitive CRT is comparable with that after esophagectomy [10, 11], one of the biggest drawbacks of definitive CRT is the higher incidence of local failures [20, 21]. Previous studies on definitive CRT have shown local failures in 19–29% of patients [9–11]. These patients sometimes required esophagectomy, which is associated with high morbidity and mortality [22].

In patients with SEC, the depth of invasion can be estimated using magnifying endoscopy [23]; however, its evaluation demands a high level of proficiency in using magnifying endoscopy. Furthermore, it is difficult to strictly distinguish T1a and T1b tumors using current endoscopic techniques. In fact, in our study, 4 of the 16 clinical T1b were pathological T1a-MM. ESD can be a diagnostic and therapeutic tool. Recent advances in ESD techniques have enabled the complete removal of T1b tumors, leading to an accurate estimation of the depth of invasion [24]. Local control rates of ESD have been reported to be > 95% [25]. However, patients with T1b tumors and/or lymphovascular invasion have a higher risk for lymph node metastasis; therefore, ESD alone cannot be considered curative in such patients [6–8].

We have previously reported surgical safety in 12 patients who underwent esophagectomy via a laparoscopic transhiatal approach out of 16 patients who underwent esophagectomy after ESD (i.e., the esophagectomy group in our study). These 12 patients had comparatively higher incidences of serious complications, including respiratory complications (3/12), recurrent nerve palsy (4/12), and anastomotic leaks (1/12) [17]. Because of the high rate of serious complications in esophagectomy, an alternative nonsurgical option was needed, which intensified CRT in the primary management after ESD for patients with T1b tumors. ESD followed by CRT would theoretically have high local curability and fewer cardiopulmonary adverse events. ESD is performed to completely remove or reduce the size of superficial tumors, thereby reducing the risk of local failures. CRT can be performed after confirming the histologic findings of cancer and estimating the risk of recurrence by examining the specimens. Because the diagnostic accuracy of endoscopy for T staging of superficial tumors has been controversial, confirming the histological findings of cancer after ESD may help avoid overtreatment in some patients with pathological T1a tumors.

We assessed patients who underwent CRT after 2014 to acquire data from uniform populations with similar radiation fields and chemotherapeutic regimens. No patients experienced locoregional failures. Although metachronous esophageal lesions were found in two patients, they were successfully treated with ESD. Thus, we confirmed that this therapeutic approach is an excellent local management method. However, our results also suggested that rigorous follow-up is necessary for these patients.

Only few studies have investigated the efficacy of ESD followed by CRT for stage I esophageal squamous cell carcinoma [9, 12, 26]. Kawaguchi et al. [9] have reported no local or distant metastasis in 16 patients with T1b or T1a-MM tumors with positive lymphovascular invasion who underwent CRT after ESD. In a recent retrospective study by Hamada et al. [12], distant metastases and local recurrences were found in 3 and 9%, respectively, of 66 patients with SEC who underwent endoscopic resection followed by CRT. Although CTV was smaller in our study than in these studies [9, 12], the clinical results seemed comparable, which might be because of our stronger chemotherapeutic regimen (5-FU 1000 mg/m^2^ on days 1–4 and 29–32 and CDDP 75 mg/m^2^ on days 1 and 29) compared with that of previous studies (5-FU 700 mg/m^2^ on days 1–4 and 29–32 and CDDP 70 mg/m^2^ on days 1 and 29). Our results also suggested that the smaller irradiation field and our chemotherapeutic regimen did not increase the risk of nodal relapse outside the irradiation field.

Adverse events, including cardiopulmonary toxicity, are also large limitations of CRT [9, 27]. In Japan, the standard radiation dose for patients with esophageal cancer receiving definitive CRT has been 40 Gy to the surrounding esophagus, followed by a 10–20 Gy coned-down boost to the primary tumor, totaling to 50–60 Gy. In patients with completely resected T1b tumors with negative resection margins, a boost irradiation of 10–20 Gy to the primary lesion could be probably omitted to reduce the adverse events.

Grade ≥ 3 hematological and nonhematological adverse events of definitive CRT have been reported in 26 and 32% of patients, respectively [20]. Hematological and nonhematological adverse events are generally mainly induced by chemotherapy and radiotherapy, respectively. In our study, the incidence of nonhematological adverse events was only 6%, which was much lower than that of previously reported definitive CRT, which may have been because of the lower radiation dose and smaller irradiation field. None of our patients experienced radiation pneumonitis. Furthermore, irradiation was applied in a four-field method to reduce the cardiac dose, which resulted in no patients experiencing adverse events of the heart.

Esophageal strictures are common adverse events of ESD [26, 28]. Treatment-related esophageal strictures were observed in two of our patients (13%), which were manageable by medication and endoscopic balloon dilatation. We could not completely evaluate late toxicity in this study because of the short follow-up period. Therefore, further follow-up and evaluation for late toxicity are needed.

In our study, no significant differences were observed in the prognosis of both groups. Combined treatment with ESD and CRT might offset their shortcomings and be less invasive than a surgical approach. To our knowledge, this is the first report directly comparing the effect of CRT and esophagectomy as an additional treatment after ESD.

Our study had several limitations, including the retrospective design, small sample size, and short follow-up period, which may have limited the statistical power. A phase II study is ongoing in Japan to evaluate the efficacy and safety of ESD followed by CRT for clinical stage I (T1bN0M0) esophageal cancer [29], and the results are expected in the near future.

## Conclusions

In conclusion, ESD followed by CRT is a safe and effective method for the treatment of patients with T1b tumors. This therapeutic approach is not inferior or, in view of the lower degree of invasiveness, potentially superior to surgical resection. Therefore, we believe that our data support the use of nonsurgical treatments in patients with T1b tumors. To our knowledge, this is the first report to directly compare the effect of CRT and esophagectomy as additional treatments after ESD.

## Abbreviations

CDDP: Cisplatin
CRT: Chemoradiotherapy
CT: Computed tomography
CTV1: Initial clinical target volume
DFS: Disease-free survival
DI: Droplet infiltration
ESD: Endoscopic submucosal dissection
FU: Fluorouracil
INF C: Infiltration pattern C
NCI-CTCAE: National Cancer Institute Common Terminology Criteria for Adverse Events
OS: Overall survival
PET/CT: Positron emission tomography/computed tomography
PTV: Planning target volume
SEC: Superficial esophageal cancer
T1a-MM: Tumor invading muscularis mucosae
T1b-SM: Tumor invading submucosa
